# Low Meat Consumption in the Netherlands Is Associated With Higher Intake of Fish, Nuts, Seeds, Cheese, Sweets, and Snacks: Results From a Two-Part Model

**DOI:** 10.3389/fnut.2021.741286

**Published:** 2022-01-26

**Authors:** Samantha N. Heerschop, Sander Biesbroek, Hendriek C. Boshuizen, Pieter van't Veer

**Affiliations:** Division of Human Nutrition and Health, Wageningen University and Research, Wageningen, Netherlands

**Keywords:** acceptability, meat consumption, diet shift, two-part model, diet transition

## Abstract

Studies on sustainable diets show a need for replacement of animal-based foods by plant-based foods, which is also called “the protein transition.” To gain insight into the acceptability of such diet shifts, this study evaluated which current food sources people consume at varying amounts of meat consumption. The study population consisted of 4,313 participants aged 1–79 years of the Dutch National Food Consumption Survey 2012–2016, which assessed diet using two nonconsecutive 24-h dietary recalls. A two-part statistical model was used that accounts for both repeated measures and the correlation between probability and amount of consumption. Results are presented for quartiles of low to high meat consumption, by age and sex. Depending on age and sex, a higher consumption of fish (>100%), nuts and seeds (73–156%), cheese (34–111%), and sweets and snacks (28–81%) is observed in the lowest quartile of meat consumption compared to the highest. For fish, nuts, seeds, and cheese, this increase is mainly due to probability of consumption (>100%, 61–93%, and 16–64%, respectively). For sweets and snacks, the increase is mainly due to the amount of consumption (26–72%). Probability of potato consumption is 29–51% lower at low meat consumption. Vegetable consumption is lower mainly due to amount of consumption (6–29%). The results from the two-part model suggest that shifting away from a traditional Dutch high meat-vegetable-potatoes pattern is associated with higher probability of consuming fish, nuts and seeds, and cheese, but also increased amounts of sweets and snacks. This illustrates that analyzing the probability and amount part separately in relation to behavioral or physiological determinants extends our understanding of the diet according to meat consumption. These insights are important when developing realistic and acceptable food-based dietary guidelines for meat reduction.

## Introduction

Current global food production and consumption is responsible for 20–30% of total greenhouse gas emissions (GHGEs) (1). To reach the Sustainable Development Goals of the United Nations by 2030, among others a large shift is required in food production methods and consumption patterns (2). Several studies modeled healthy and sustainable diets, proposing alternative diets for specific populations (3–5). Overall, these modeled diets show a replacement of animal-based foods by plant-based foods, also called “the protein transition” (6). The EAT-Lancet commission has proposed a healthy and sustainable reference diet that enables to feed the world within its planetary boundaries (7). This reference diet indeed requires a clear transition to plant-based foods as it includes high consumption of vegetables, fruit, whole grains, legumes, nuts, and unsaturated oils, moderate consumption of seafood, dairy, and poultry, and minimal consumption of red meat, processed meat, added sugar, refined grains, and starchy vegetables.

Of animal-based products, meat and especially beef provides the largest share in environmental impact (8, 9). Furthermore, long-term consumption of increasing amounts of red and processed meat is associated with total mortality, cardiovascular disease, colorectal cancer, and type 2 diabetes in both men and women (10, 11). Both the environmental impact and the negative health effects that are related to a high red and processed meat consumption provide a wide consensus on the need to decrease meat consumption (3, 5). As meat is a major protein source, it is important to replace these proteins both quantitatively and qualitatively (12). Furthermore, for consumers to accept and implement healthy and sustainable diets, and for governments to create policies, it is important that replacement of certain products such as meat is affordable, reliable, and acceptable to consumers. Modeled healthy and sustainable diets often take acceptability into account as “distance from current diet” (9, 13, 14). However, these modeled diets still deviate substantially from current dietary patterns, which cause both policy makers and food companies to question the acceptability of these diets. Therefore, it is necessary to gain more insight into substitutions that occur in the diet when comparing high and low meat consumption.

Analyzing and describing dietary patterns is faced with several statistical challenges. Distributions of food consumption data are often skewed, partially because of occasionally consumed foods. In addition, a relationship may exist between the probability of consumption and the amount of consumption at such an occasion, for example, those consuming a product more often may consume larger amounts of this product as well. Two-part mixed-effects models can solve these problems by splitting the data into zero and nonzero values, while taking into account the correlation between probability of consumption and the amount consumed (15–17). However, in food consumption surveys, food intake is often measured on multiple days. A common standard is to use at least two independent days (18). As the standard two-part model does not take repeated measures into account, Tooze et al. (19) proposed a mixed distribution model that takes into account both repeated measures and the correlation between probability and amount of consumption (19).

To get insight into acceptable substitutions for meat in the diet, this study analyzed dietary patterns to describe what food groups are consumed instead of meat in subgroups of the Dutch population, using data from the Dutch National Food Consumption Survey 2012–2016 (DNFCS) (20).

## Materials and Methods

### Study Population

The study population consisted of participants of the DNFCS (20). This survey consisted of 4,313 participants aged 1–79 years and was conducted between 2012 and 2016 by the National Institute for Public Health and the Environment (RIVM), the Netherlands. Participants were drawn from a representative Dutch consumer panel of the market research agency KANTAR TNS. Panel members participate in all types of studies. An age-gender random sampling strategy was applied. Representativeness of region, address density, age groups, and education was taken into account. Institutionalized people, people in hospital or who were terminally ill, tube-fed, or parenterally fed people, women who were pregnant or breastfeeding, persons who participated in a national food consumption survey during the past 4 years, people with inadequate command of the Dutch language, and persons who were otherwise incapable of participating in a food consumption survey (e.g., persons who are deaf) were excluded from participation. The DNFCS was conducted according to the guidelines of the Declaration of Helsinki. This study was deemed exempt by the Utrecht University Medical Ethical Review Committee (Institutional Review Board) because the study was not subjected to the Medical Research Involving Human Subjects Act (WMO) of the Netherlands (reference number 12-359/C). In line with this, written informed consent was not required according to the regulations at the time of data collection.

### Dietary Assessment

Trained dieticians collected food consumption data by two nonconsecutive 24-h dietary recalls for people aged 9–70 years. Standardized interviews were conducted using the GloboDiet (former EPIC-soft©) computer program, provided by the International Agency for Research on Cancer, Lyon, France (21). For children aged < 9 years, diet was assessed by means of the dietary record method for two nonconsecutive days. Usually the next day, the diet record was followed by a completion of interview similar to the 24-h recall. A caretaker was involved, together with the child. Participants aged > 70 years received an additional food recording booklet to be kept at the day before the call. To obtain consumption information independent of possible fluctuation in dietary patterns per season, the 24-h dietary recalls were spread over seasons and days of the week, both week and weekend days.

Food composition of the consumed products was derived from the Dutch Food Composition Database to calculate intake of energy and nutrients (NEVO-online version 2016/5.0) (22). Food items from the DNFCS were grouped into 29 food groups adapted from the GloboDiet food group categorization. Supplementary Material 1 shows detailed information about the products included in each of these food groups. Consumption is expressed as g/2,000 kcal. Explanation on measurements on lifestyle and anthropometric variables are described elsewhere (20).

### Statistical Analysis

Participants were categorized into children (aged 1–8 years), teenagers (aged 9–18 years), adults (aged 19–50 years), and older adults (aged 51–79 years) to stratify for differences in overall food consumption and specific food sources that replace meats according to age. In Table 1, continuous variables are displayed as mean ± standard deviation (SD) and discrete variables as percentages. All analyses were stratified for age, using four abovementioned age groups. The statistical analysis was performed using SAS software, version 9.4 (SAS Institute Inc., Cary, NC, USA).

**Table 1:** Population characteristics, energy intake (kcal), energy density (kcal/100 g), animal- and plant-based food intake (g/2,000 kcal), and macronutrient intake (g/2,000 kcal) per age group.

	**Age group**			
	**1–8 years (children)**	**9–18 years (teenagers)**	**19–50 years (adults)**	**51–79 years (older adults)**
*N*	1.192	1.043	1.039	1.039
Male (%)	49.7	50.7	50.0	50.4
Age (years)	3.8 ± 2.3	13.4 ± 2.7	33.5 ± 9.5	67.5 ± 8
Height (cm)	104.4 ± 17.8	163.3 ± 14.4	176.5 ± 9.8	173.7 ± 10.3
Weight (kg)	18.2 ± 6.5	54.5 ± 15.4	79.4 ± 16.4	83.1 ± 15.6
BMI (kg/m^2^)	-^a^	20.1 ± 3.5	25.5 ± 4.9	27.9 ± 5.1
Energy intake (kcal/day)	1.405 ± 418	2.124 ± 739	2.277 ± 883	2.056 ± 666
Energy intake (kcal/day)^b^				
Meat quartile one	1,238 ± 417	1,896 ± 652	1,979 ± 767	1,849 ± 668
Meat quartile two	1,276 ± 374	2,008 ± 758	2,154 ± 826	1,921 ± 624
Meat quartile three	1,325 ± 383	1,931 ± 610	2,079 ± 752	1,898 ± 572
Meat quartile four	1,336 ± 376	1,840 ± 646	1,974 ± 738	1,808 ± 570
Energy density (kcal/100 g)^b^				
Meat quartile one	143 ± 43	192 ± 57	174 ± 60	162 ± 53
Meat quartile two	144 ± 43	185 ± 56	180 ± 54	160 ± 45
Meat quartile three	147 ± 41	181 ± 52	172 ± 49	155 ± 43
Meat quartile four	146 ± 40	184 ± 53	172 ± 54	149 ± 41
Food intake in g/2,000 kcal				
Solid plant-based^c^	723 ± 280	652 ± 235	735 ± 340	807 ± 330
Solid animal-based^c^	222 ± 164	212 ± 141	254 ± 172	304 ± 160
Of which meat	65 ± 53	89 ± 70	92 ± 75	98 ± 74
Milk	434 ± 353	215 ± 242	177 ± 238	176 ± 204
Drinks excluding milk^c^	999 ± 614	1.339 ± 796	2.131 ± 1.392	1.991 ± 1.138
Macronutrient intake in g/2,000 kcal				
Carbohydrates	272 ± 39	253 ± 40	225 ± 47	208 ± 43
Fat	67 ± 16	75 ± 17	78 ± 19	79 ± 17
Protein	66 ± 15	67 ± 19	77 ± 22	80 ± 21
Animal protein	38 ± 16	39 ± 19	46 ± 23	51 ± 22
Plant protein	27 ± 8	28 ± 8	30 ± 9	29 ± 8

*Data derived from the Dutch National Food Consumption Survey 2012–2016*.

*Mean and standard deviation are shown for continuous variables*.

*Categorical variables are presented as percentages*.

a *BMI is not a valid measure for body fat in children and therefore not shown in this age group (23)*.

b *Based on solid foods and milk, including meat*.

c *Food groups belonging to solid plant-based, solid animal-based, and drinks are depicted in Supplementary Material 1*.

#### Two-Part Model

Due to the repeated measures and the clumping at zero and thereby extreme nonnormality of the data, a mixed-effects mixed distribution model with correlated random effects of probability and amount was used. The two 24-h dietary recalls per participant were not averaged as the aim of this study was to get insight into frequency and amount of consumption, which effects would be attenuated when averaging the food consumption data. The two-part model contained a logistic part to model the probability (i.e., binomial component) of a nonzero value and a log-normal part to model the distribution of nonzero values, allowing for repeated measurements using random effects and allowing for correlation between the random effects of probability and amount. Such a model was created in a SAS macro by Tooze et al. (24) (MIXCORR, available from the authors) (19). In each of the four age subgroups, the dependent variable was any of the 29 food groups, such as potatoes, vegetables, etc. The amount part of the dependent variable was assumed to be log-normal distributed. Covariates for the binomial and also for the log-normal component of the model were total meat consumption (continuous variable), sex, and an interaction term of total meat and sex. The article of Tooze et al. provides more detailed information on the application of this model (24).

#### Predictions

**Figure 1:**
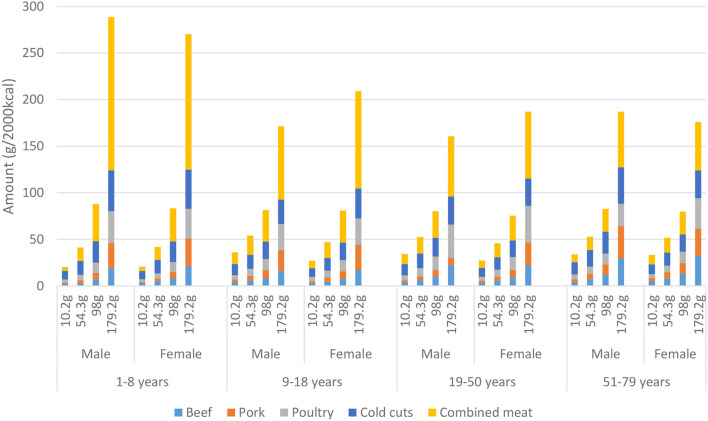
Consumption per type of meat presented by mean total meat consumption per quartile (based on the total population), gender and age derived from the Dutch National Food Consumption Survey 2012–2016.

Predicted intakes per food group were calculated for the mean of the total meat consumption per quartile (see Figure 1 for mean total meat consumption per quartile). Meat quartiles were based on consumption in g/2,000 kcal for the total population. The two-part model was not applied for defining meat quartiles. To calculate a predicted value, first 10,000 random effects for the probability model (u1v) and the amount model (u2v) were randomly drawn based on the covariance matrix of u1v and u2v. This way correlation between u1v and u2v is taken into account. Next, the predicted log amount and log odds were obtained using the parameter estimates from the model and adding the values for the random effects (u1v and u2v), for men and women separately. To back transform the values for amount, the exponent of the log amount was taken, and 0.5 times the variance of the day-to-day variation (within-person variation) was added (25). To back transform the values for the odds, the exponent of the log odds was taken. The probability was calculated as odds/(1+odds). Finally, the predicted intake was obtained by multiplying the probability by the amount. Then, the average of the 10,000 simulated predicted intakes was taken. This average represented the predicted value for a person who consumes the mean amount of meat within a specified quartile. Supplementary Material 2 shows a diagram of these steps. The syntax including formulas and additional explanation can be found in Supplementary Material 3. Predicted values should be interpreted as follows: on a day that a person consumes a given (high or low) amount of meat, a group of such persons would on average consume this predicted amount of food group X, integrating the predicted probability and amount. The association between the observed meat consumption on a specific day and the average consumption of food groups X therefore originates from the product of the consumption probability and amount of X, each of them predicted by the separate models for probability and amount.

For a few food groups, the covariance between the error terms could not be estimated (see Supplementary Material 4). These were food groups that were consumed nearly every day by all subjects or food groups that were hardly consumed on two days by any subject. The spread of the data was insufficient for the model to estimate the correlation between probability and amount. For these food groups, an uncorrelated model was used to calculate predicted values. A similar procedure as described above was used, but now u1v and u2v were drawn independently of each other.

#### Confidence Intervals for Predictions

To calculate the confidence intervals randomly, 1,000 new sets of parameter estimates were drawn using the model parameter estimates and their covariance matrix from the model that was used (correlated or uncorrelated). Then, the steps from the paragraph “Predictions” were repeated for all 1,000 sets of parameter estimates using a loop in SAS yielding 1,000 new predicted values. Finally, the 2.5 and 97.5 percentile were taken to obtain the confidence interval of the predicted values.

## Results

### Population Characteristics

Table 1 shows that body mass index (BMI) tends to increase with age. Total energy intake of solid foods and milk, including meat, is similar over the meat quartiles. Also, energy density remains similar over the meat quartiles (beverages other than milk excluded). Both meat consumption and solid animal-based food tend to increase with age. A decreased milk consumption with increased age was observed. Energy-standardized fat and protein consumption tend to increase with age. The increase in protein consumption is mainly attributable to the increase in animal protein consumption, as plant protein consumption is similar among all age groups.

### Meat Consumption

Figure 1 shows that meat consumption increases in subsequent meat quartiles for both sexes and all age groups. Children (aged 1–8) in quartile four consume 50 to 60% more meat in gram per 2,000 kcal compared to older age groups in this quartile. In the lowest quartile of meat consumption, the share of combined meat amounted 20–35% and increased to 29–57% in the highest quartile. Cold cuts ranged from 23–45% in the lowest quartile to 15–21% in the highest quartile. An increased share of beef, pork, and poultry was observed when age increased, that is, 28–35% in the youngest and 36–53% in the oldest age group.

### Average Consumption per Food Group

**Figure 2:**
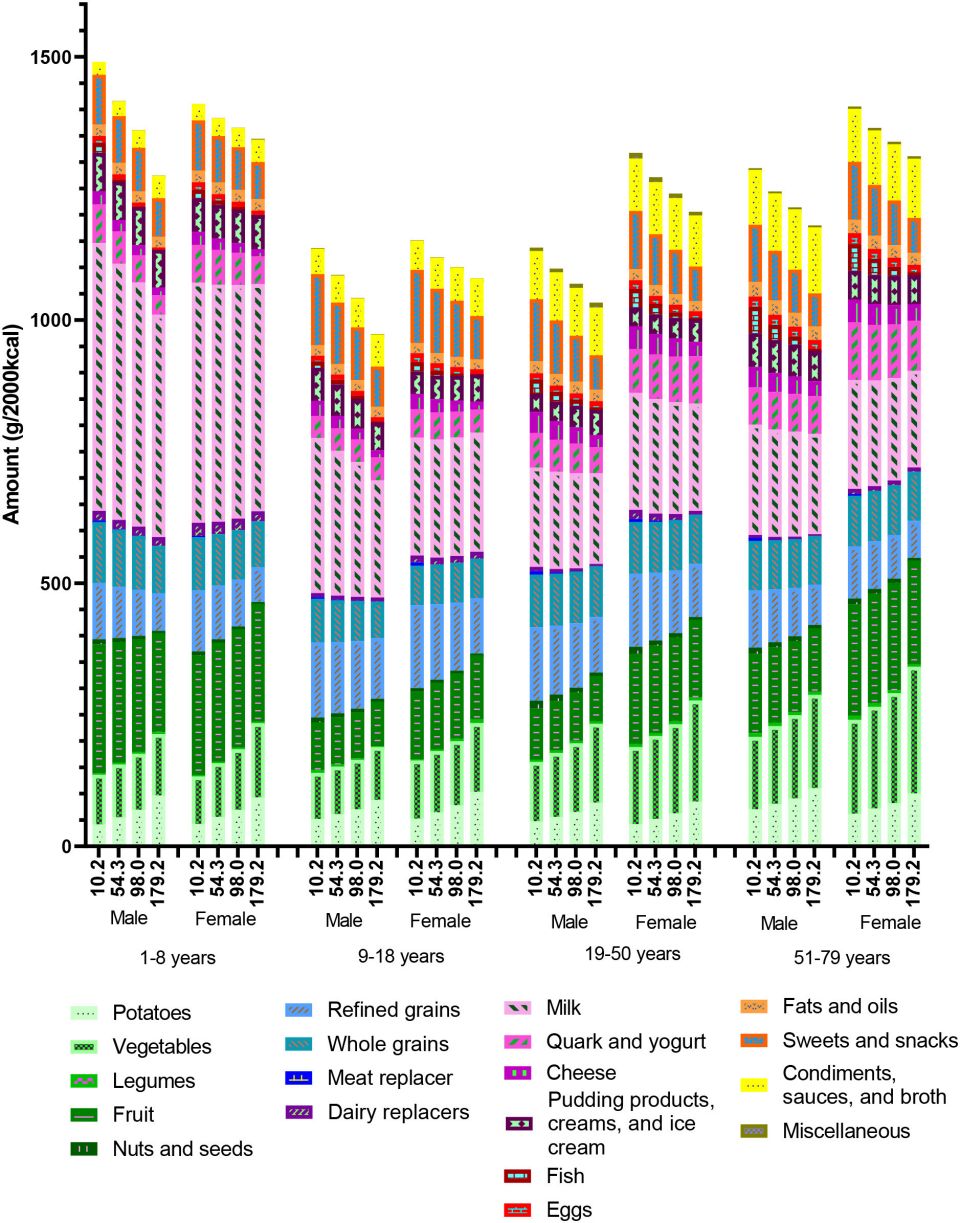
Predicted quantity (g/2,000 kcal) per solid food group and milk, excluding other beverages and meat in the observed diets in the Dutch National Food Consumption Survey 2012–2016, presented by mean total meat consumption per quartile, gender, and age. Means and 95% confidence intervals on which this figure is based can be found in Supplementary Material 4.

Figure 2 shows that the total amount of solid foods and milk, excluding meat, per 2,000 kcal, tends to increase with decreasing meat consumption. This shows substitution of food products in the dietary pattern when meat consumption decreases. Potato consumption decreases with 37–57% and vegetable consumption with 13–36% when comparing meat quartile one with quartile four. Relative milk consumption is about two times as high in children as in older age groups when expressed per 2,000 kcal. Furthermore, men between 9 and 18 years show higher milk consumption when meat consumption is low. Quark and yogurt consumption is slightly higher in women then in men between the age of 19–79. Refined grains, cheese, and sweets and snacks consumption are higher when meat consumption is low for all age and sex groups with 24–76%, 34–111%, and 28–81%, respectively. Consumption of condiments, sauces, and broth tends to increase with increasing age. Means and 95% confidence intervals on which this figure is based can be found in Supplementary Material 4.

### Association With Meat Consumption

**Figure 3:**
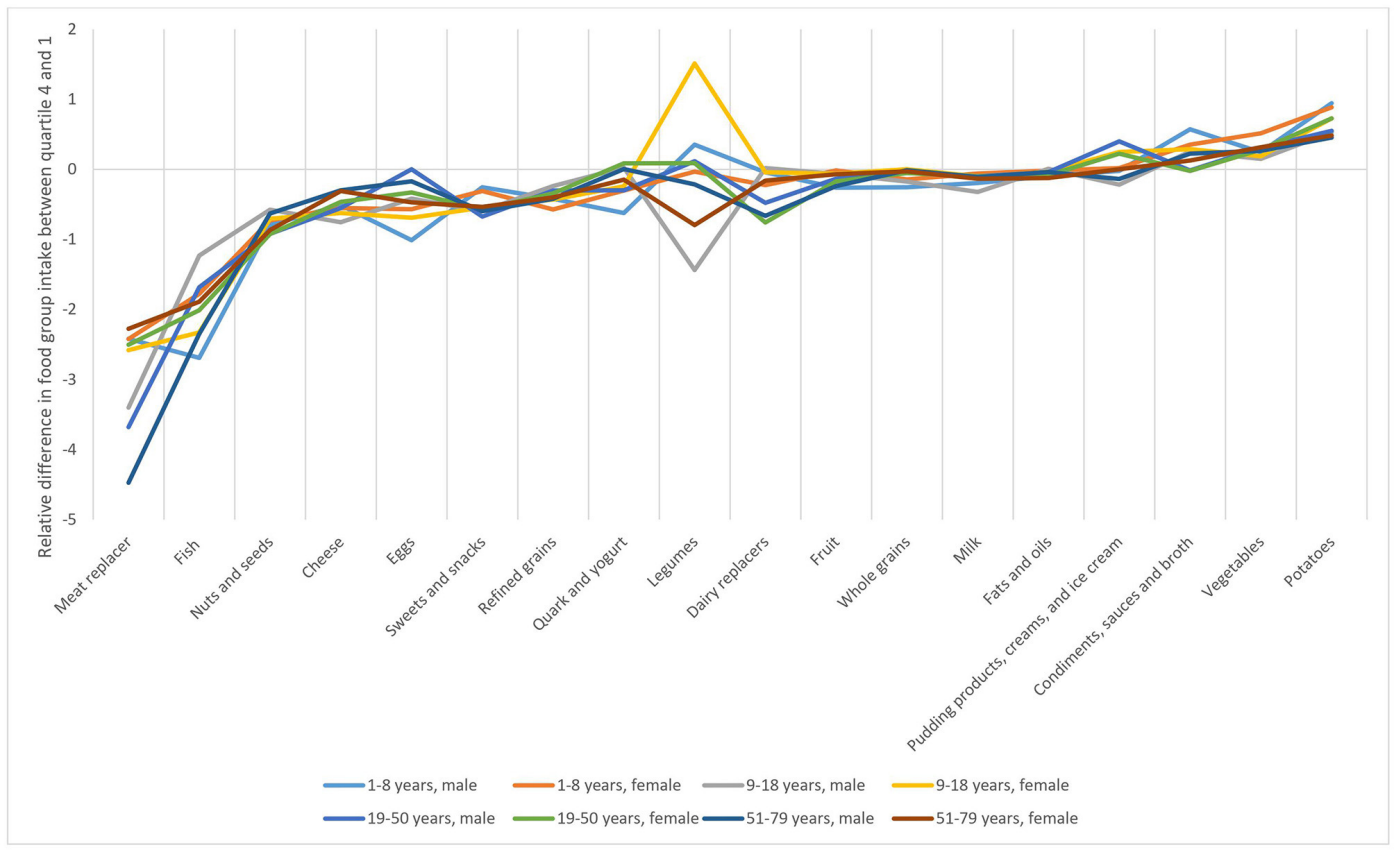
Difference in predicted quantity (g/2,000 kcal) between the highest and lowest quartile of meat consumption, divided by the mean of the corresponding age and gender group, per solid food group, and milk in the observed diets in the Dutch National Food Consumption Survey 2012–2016, stratified by age, corrected for sex and the interaction between sex and meat consumption.

Figure 3 shows the relative difference in consumption between quartiles four and one for solid food groups and milk, excluding miscellaneous, for all age and sex groups separately. The relative difference was calculated as the difference in predicted quantity (g/2,000 kcal) between the highest and lowest quartile of meat consumption, divided by the mean of the corresponding age and gender group, per solid food group, and milk. For example, for fish in the subgroup men aged 51–79 years, the mean predicted consumption of quartile four was 4,22 g/2,000 kcal, and in quartile one, this was 49,97 g/2,000 kcal. The average consumption for men aged 51–79 years was 19,50 g/2,000 kcal. The relative difference in predicted quantity was then (4,22–49,97)/19,50. Meat replacers, fish, nuts and seeds, and cheese are relatively strongest negatively associated with meat consumption, which means that if meat consumption decreases, consumption of the respective food group increases. Vegetables and potatoes are strongest positively associated with meat consumption. Quark and yogurt, legumes, dairy replacers, fruit, whole grains, milk, fats and oils, and pudding products, creams, and ice creams are not associated with meat consumption according to the data. Of these food groups, meat replacers, legumes, and dairy replacers were consumed by only a limited number of subjects (2–8% of the population).

### Specific Food Groups

**Figure 4:**
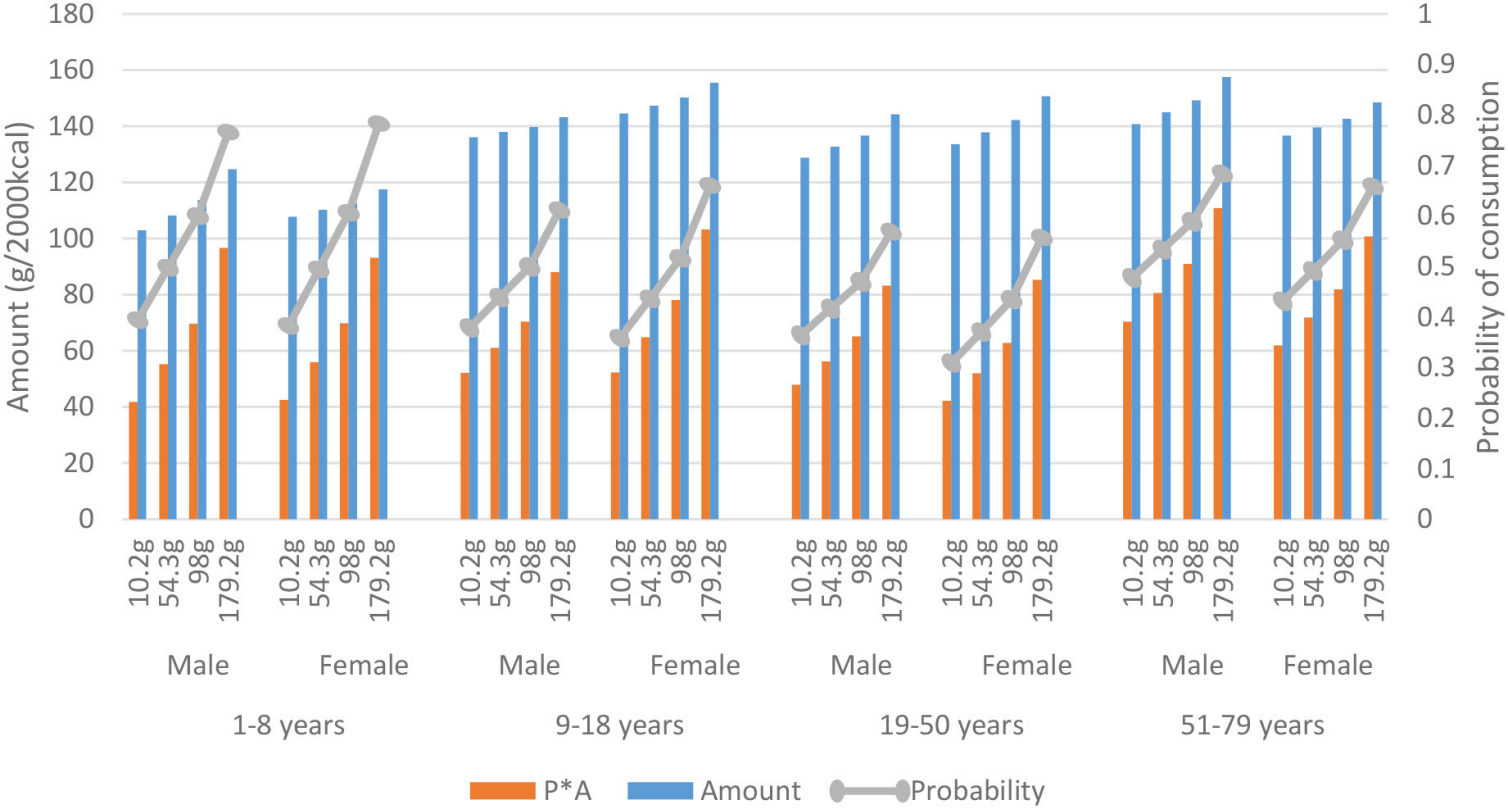
Consumption of potatoes presented by mean total meat consumption per quartile, gender, and age. The predicted quantity (P*A) (g/2,000 kcal) is the modeled amount among users (Amount), times the modeled probability of consumption (probability) derived from the Dutch National Food Consumption Survey 2012–2016.

The following three figures highlight food groups for which a trend with meat consumption was observed. These food groups are highlighted based on their variation in either probability or amount causing the observed trend with meat consumption. First, Figure 4 shows that the probability of potato consumption is on average (of all age and sex groups) about 0.45 in the lowest meat quartile, which means that potatoes are consumed on ~3 days of the week (0.45 ^*^ 7 (days) = 3.15). In the lowest meat quartiles, the probability of consumption is 29%−51% lower compared to the highest meat quartiles. In children, the increase in probability of potato consumption is larger than in the older age groups. Amount of potato consumption is 5% to 17% lower at low meat consumption compared to meat quartile four. The predicted quantity (Probability ^*^ Amount) (g/2,000 kcal) is the modeled amount among users (Amount), times the modeled probability of consumption (probability). The product shows an even lower consumption of potatoes (36–57% lower) in the lowest meat quartiles. Also, vegetable consumption is lower when meat consumption is low (Supplementary Material 5). Probability of vegetable consumption is 1–16% lower at low compared to high meat consumption. Amount is 6–29% lower at low meat consumption.

**Figure 5:**
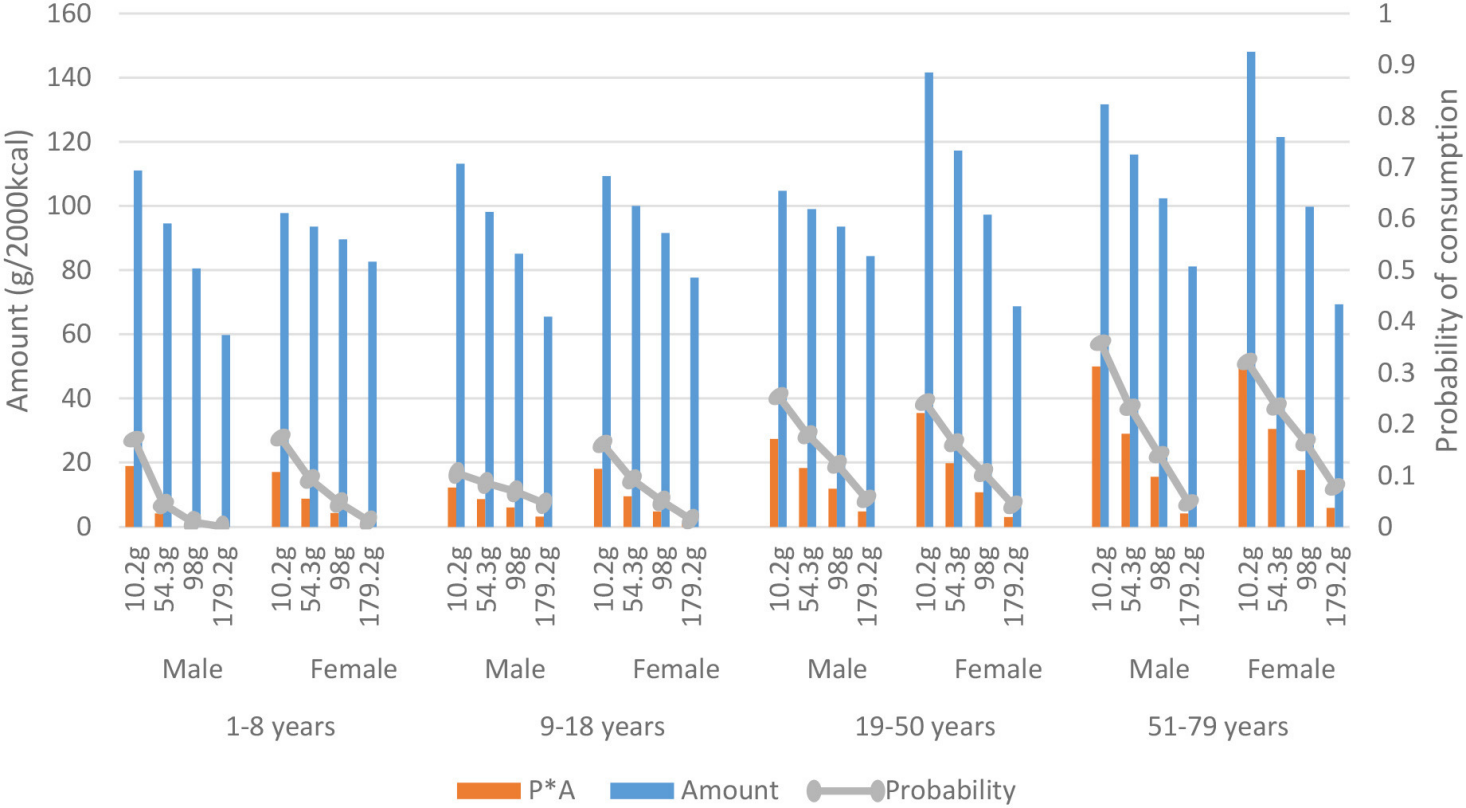
Consumption of fish presented by mean total meat consumption per quartile, gender, and age. The predicted quantity (P*A) (g/2,000 kcal) is the modeled amount among users (Amount), times the modeled probability of consumption (probability) derived from the Dutch National Food Consumption Survey 2012–2016.

Figure 5 shows that amount of fish consumption is 24–113% higher at low compared to high meat consumption. As frequency of fish consumption was very low in the highest meat quartiles, the ratio of probability of fish consumption in meat quartiles one to four is very high. Probability of fish consumption in meat quartile one is 2.2 to 17 times as large as compared to meat quartile four, showing the replacement of meat by fish within a meal. Overall, fish consumption is >100% higher when meat consumption is low.

**Figure 6:**
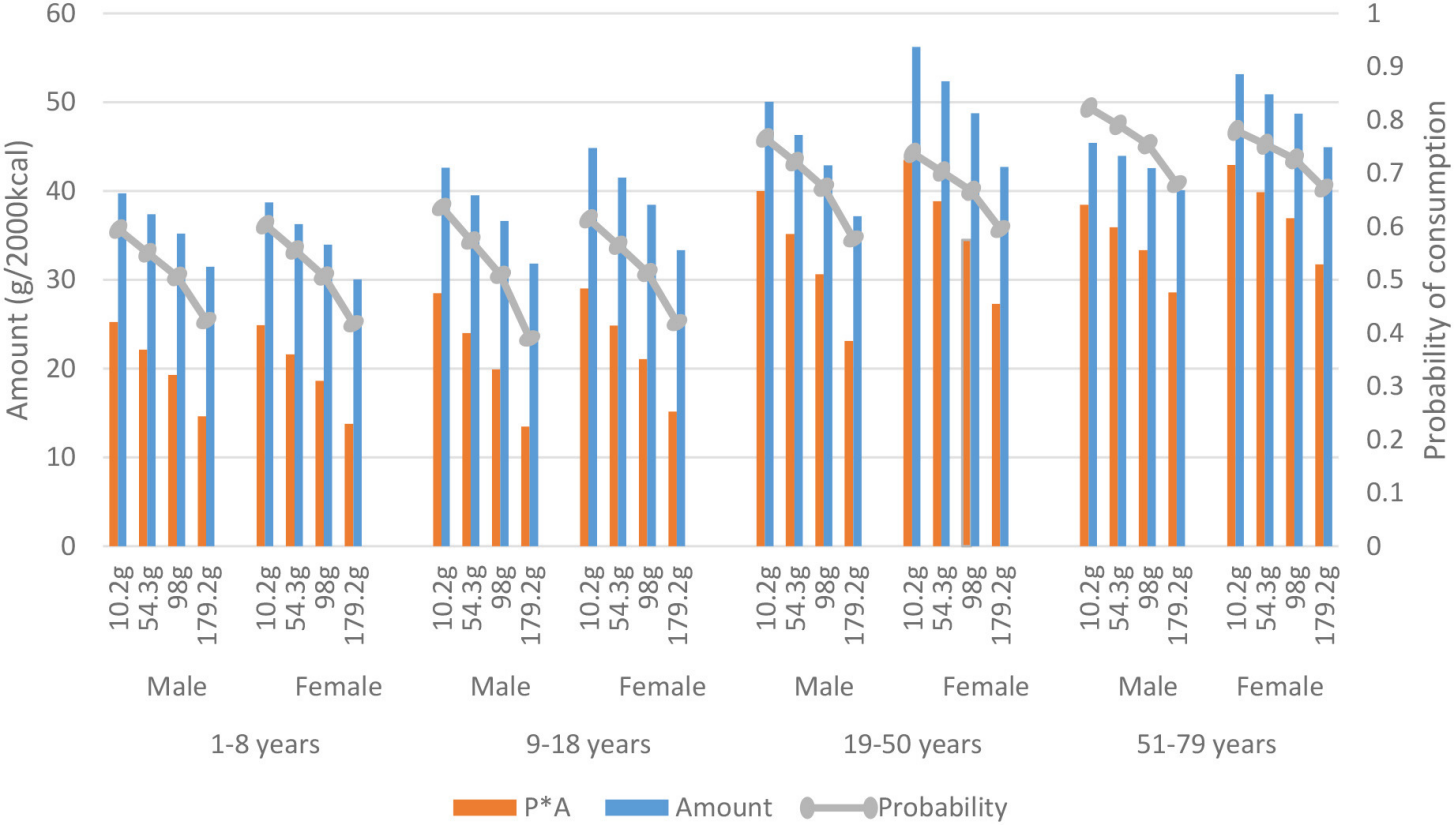
Consumption of cheese presented by mean total meat consumption per quartile, gender, and age. The predicted quantity (P*A) (g/2,000 kcal) is the modeled amount among users (Amount), times the modeled probability of consumption (probability) derived from the Dutch National Food Consumption Survey 2012–2016.

Figure 6 shows that cheese consumption is higher at low meat consumption for all age groups and both sexes (34–111% higher). Probability is 16–64% higher at low compared to high meat consumption, whereas amount is 13–37% higher. Similar trends are observed for the food groups nuts and seeds (73–156% higher) and refined grains (24–50% higher) (Supplementary Materials 4, 5B,C). For nuts and seeds, probability is 61–93% higher when meat consumption is low. Amount is −3–53% higher at low compared to high meat consumption. Consumption of refined grains is 10–38% and 10–33% higher at low meat consumption, respectively. Consumption of sweets and snacks increases with 28–81% when meat consumption decreases (Supplementary Materials 4, 5D). This increase is mainly due to a higher amount of consumption (26–72% higher at low compared to high meat consumption) and not to probability of consumption (2–8% higher at low compared to high meat consumption).

Supplementary Materials 4, 5E show that whole grain consumption increases with decreasing meat consumption in children. For the other age groups, whole grains is not associated with meat consumption.

## Discussion

This study evaluated consumption of food groups for varying amounts of meat consumption for the Dutch National Food Consumption Survey (2012–2016). Results show that, depending on age and sex, a higher consumption of fish, nuts and seeds, cheese, and sweets and snacks is observed in the lowest quartile of meat consumption compared to the highest. For fish, nuts and seeds, and cheese, the higher consumption is mainly due to probability of consumption, pointing at compensation of lowered meat consumption by these food groups. For sweets and snacks, the higher consumption is mainly due to the amount consumed. In the lowest meat quartile, probability of potato consumption is lower compared to the highest quartile. Vegetable consumption in meat quartile one is lower mainly due to amount. In the highest meat quartile, the results show a typical characteristic of the Dutch dietary pattern with a traditional dinner consisting of potatoes, vegetables, and meat. Less traditional meals and lower meat consumption go together with an increase in refined grains, which probably reflects substitution of potatoes by refined pasta or white rice.

In the Dutch eating pattern, breakfast and lunch often consist of bread with sweet and/or savory toppings. As the increased amount of cheese consumption at low meat consumption is about 10 g/2,000 kcal, this suggests that cold cuts are replaced by cheese. The same applies for nuts and seeds. Peanut butter is classified in this food group, and bread with peanut butter typically substitutes bread with cold cuts. The higher consumption of sweets and snacks when meat consumption is low is mainly due to the amount of consumption and only barely to the probability, implying that sweets and snacks are even frequently consumed, but in larger portions. Apart from substitutions within meals, substitution can also occur over a day, between meals. People who consume large amounts of meat as part of their meals may experience higher satiety and consume a smaller amount of sweets and snacks in-between meals. This is supported by literature that shows that savory dishes and meat are perceived as more satiating food items than plant-based food (26). However, literature also shows that animal- vs. plant-based protein foods have no different effect on satiety in healthy adults (27).

It should be noted that sweets and snacks is a food group that contains a large variety of products. As a result, almost everyone consumes at least something in that food group every day, leading to a consumption probability close to one for the day as a whole. Besides, aggregating a diversity of foods into a single food group can mask relevant associations. Therefore, a similar analysis that accounts for meal moments could provide useful additional insight. It can be concluded that compensation for meat consumption is isocaloric as both energy intake and energy density remain stable over the meat quartiles. These results suggest that separating average food consumption into a “probabilities” and “amounts” part can help to understand the relation of diet substitution patterns to cultural preferences (e.g., savory/sweet toppings) and regulatory physiological mechanisms (e.g., satiety, energy balance).

Diet models have been used to study trade-offs between different strategies toward healthy and sustainable diets (13). Such diet models make use of linear combinations of either foods or whole diets to maximize nutrient contents and to minimize environmental impact. To operationalize acceptability of dietary patterns, such models use measures of deviation from the observed average food consumption (28, 29). Modeling studies that use linear combinations of foods may result in large deviations from the current diet or less diversity of foods and therefore require additional constraints to preserve acceptability (29). A different approach was used in a study of four European counties (Denmark, Czech Republic, Italy, and France) (14). This study used linear combinations of whole diets that were first benchmarked by means of food-based dietary guidelines. This partially preserves implicit associations between food groups and the resulting dietary patterns remained within the range of observed diets. Nevertheless, in all diet models, the metrics for acceptability of dietary patterns are based on the consumption of a(n) (averaged) day for an individual or a population. Dietary patterns, however, consist of foods that are used in certain frequencies and amounts with interdependencies between these two and between other foods. The two-part model used in this study is a first attempt to separate out these different elements and may be of help to arrive at metrics for acceptability of dietary shifts that account for cultural and physiology-based substitution patterns in an improved manner. The present observational study shows what substitutions or eliminations occur in practice, which could eventually help to evaluate whether solutions from diet models are achievable for a population. A study that used quadratic optimization to model Dutch diets that satisfied both nutritional goals and GHGE targets found that beef, pork, cheese, snacks, and butter consumption should decrease with more than 33% compared to their baseline. Legumes, fish and shellfish, peanuts, tree nuts, vegetables, soy foods, and soy drink should increase with more than 150% compared with baseline (29). For fish and nuts and seeds, our results are in line with this diet-modeling study. However, in the diet-modeling study, cheese, sweets and snacks, and vegetable consumption decrease with decreased meat consumption, which is opposite to the observations in this study. These insights are useful to improve diet models or to incorporate in food-based dietary guidelines that are developed with the help of such models.

Strengths of the study are the methodology to analyze dietary patterns, taking into account correlation between probability and amount of consumption and repeated measures. This model shows the least bias when compared to other methods that estimate usual intake using 24-h recall data (24). The two-part model gave insight into substitutions for meat (probability) and also the diet pattern (amount). As this study stratified for age and gender and used food groups, results were close to the behavior and food habits of the population. This analysis reveals currently observed and apparently acceptable compensations for meat in the diet. Interpretation of these results is also closer to reality than when using other methods to describe dietary patterns, for example, factor analysis, clustering methods, or principal component analysis (30, 31). Another strength is that food consumption information was based on two nonconsecutive 24-h dietary recalls. This method provides detailed food consumption information and is less subjected to bias than food frequency questionnaires (32). Finally, the used food consumption information from the DNFCS represents the most recent and representative diets of Dutch inhabitants, accounting for age, gender, region, address density, and level of education.

Also, some limitations should be mentioned. This two-part model analyzed only one food group at the time, whereas intake of different food groups is highly correlated. Furthermore, next to age and sex, covariates such as social economic status may influence food intake. As application of the current model is relatively new, we started with a single variable two-part model including straightforward covariates. The addition of the two-part model was already an improvement over the usually applied one-part model. In the future, it would be valuable to investigate whether it is possible to extend the two-part model to a multivariate model with additional covariates. The second limitation is about the variation in the number of days a product is consumed that the two-part model needs to obtain a covariance table for calculating the random effects. For a few food groups, this variation was not sufficient. In these cases, the covariance was set to zero and the model could run, disregarding the correlation between probability and amount. For food groups that had <5% zero consumptions (e.g., sweets and snacks), a two-part model was not necessary. In contrast to most food groups, the two-part model was not of added value for frequently consumed foods. However, it was insightful that in these cases, mainly the amount differed per meat quartile and not the frequency. For comparability of the results, the same model was applied to all food groups. Besides, as only two 24-h dietary recalls were used, the probability might be hard to estimate. Therefore, for occasionally consumed foods, it would be desirable to add food frequency questionnaire information as covariate, as this might improve estimating the probability of consumption (33). Furthermore, there may be misreporting in the self-reported recalls (32, 34). However, assuming that misreporting is independent of specific food groups, due to the standardization for energy intake, part of the misreporting is corrected (35). Another drawback is that classification of foods into food groups can mask relevant associations. For instance, the food group sweets and snacks contains some meat snacks such as sausage rolls and other meat pastries. These meat products are not taken into account in the total meat food group as this is a combined product. This was the case for 13 participants. As this is a small number, the impact on the results will be small.

Concluding, the results from the two-part model suggest that a shifting away from a traditional Dutch high meat-vegetable-potatoes pattern is associated with increased probability of consuming fish, nuts and seeds, and cheese, but also increased amounts of sweets and snacks. These results illustrate that analyzing the probability and amount part separately in relation to behavioral or physiological determinants would extend our understanding of the diet according to meat consumption. These insights are important when developing realistic and acceptable food-based dietary guidelines for meat reduction.

## Data Availability Statement

Publicly available datasets were analyzed in this study. This data can be found here: Dutch National Food Consumption Survey 2012–2016: https://www.rivm.nl/en/dutch-national-food-consumption-survey/data-on-request.

## Ethics Statement

Ethical review and approval were waived for this study as the Utrecht University Medical Ethical Review Committee evaluated that the study was not subject to the Medical Research Involving Human Subjects Act (WMO) of the Netherlands (reference number 12-359/C). Written informed consent for participation was not required for this study in accordance with the national legislation and the institutional requirements.

## Author Contributions

SH did the data curation, formal analysis, visualization, and wrote the original draft. SH and HB were responsible for the methodology. SB and PV acquitted the funding and supervised the research. SB, PV, and HB reviewed and edited the manuscript. All authors read and approved the final manuscript.

## Funding

This work was supported by a grant from the Dutch Dairy Association (NZO): SHARP-next seeding project (contract 2020–2021). The NZO did not play a role in the execution of the study and in the interpretation of the results.

## Conflict of Interest

PV received speakers' honorarium for: YINI-sponsored meetings at ASN (Jun 2, 2021) and in Barcelona (live Zoom session, Dec 10, 2021). The remaining authors declare that the research was conducted in the absence of any commercial or financial relationships that could be construed as a potential conflict of interest.

## Publisher's Note

All claims expressed in this article are solely those of the authors and do not necessarily represent those of their affiliated organizations, or those of the publisher, the editors and the reviewers. Any product that may be evaluated in this article, or claim that may be made by its manufacturer, is not guaranteed or endorsed by the publisher.

## Supplementary Material

Supplementary Materials 1, 3, and 5 can be found in ‘data sheet 1.docx'; Supplementary Material 2 can be found in ‘presentation 1.pptx'; and Supplementary Material 4 can be found in 'data sheet 2.xlsx.
