# Supplementary material for: Low Meat Consumption in the Netherlands Is Associated With Higher Intake of Fish, Nuts, Seeds, Cheese, Sweets, and Snacks: Results From a Two-Part Model
Source: Front Nutr. 2022 Jan 26;8:741286. doi: 10.3389/fnut.2021.741286 (PMC8825789; doi:10.3389/fnut.2021.741286)
Supplement: Supplementary file 1 [file Data_Sheet_1.docx]

Supplementary Material

# Supplement 1

Categorization of 29 food groups adapted from GloboDiet, based on the Dutch National Food Consumption Survey.

| **Food group** | **GloboDiet groups** | **Explanation type of product within food group** |
| --- | --- | --- |
| Animal based foods | | |
| Beef | ’07-01-01’ Beef |  |
| Pork | ’07-01-03’ Pork |  |
| Poultry | ’07-02’ Poultry | Chicken, hen  Turkey, young turkey |
| Combined and other hot meat | '07-00' Unclassified and combined meat and meat products  '07-01-00' Unclassified, mixed and other mammals  '07-01-02' Veal  '07-01-04' Mutton/lamb  '07-01-05' Horse  '07-01-06' Goat  '07-01-07' Rabbit  '07-02-00' Unclassified and other poultry  '07-02-03' Duck  '07-03' Game  '07-04-00' Unclassified processed meat  '07-04-01' Hot processed meat  '07-05' Offals |  |
| Cold cuts | '07-04-02' Cold processed meat |  |
| Meat replacer | ’07-06’ Meat substitutes  ’17-01’ Vegetarian products/dishes | Unclassified meat substitutes  Hot meat substitutes  Cold meat substitutes |
| Milk | ’05-01’ Milk, milk beverages and fermented milk beverages | Unclassified or combined milk and milk beverages  Non fermented milk and milk beverages  Fermented milk, milk beverages and yogurt |
| Quark and yogurt | ’05-03’ Yogurt  ’05-04’ Fromage blanc, petits suisses (quark) |  |
| Cheese | ’05-05’ Cheeses (including spread cheeses) |  |
| Other dairy products | ’05-00’ Unclassified and mixed dairy products  ’05-06’ Cream desserts, puddings (milk based)  ’05-07-00’ Unclassified creams  ’05-07-01’ Dairy creams and creamers  ’05-08-00’ Unclassified, combined ice creams/sorbets  ’05-08-01’ Ice cream (milk based) |  |
| Dairy replacers | ‘05-02’ Milk substitutes and milk substitute products  ‘05-07-02’ Non-dairy creams and creamers  ’05-08-02’ Ice cream substitutes  ’05-08-03’ Sorbet/water ice |  |
| Fish | ‘08’ Fish, shellfish and amphibians | Unclassified and combined fish products  Fish  Crustaceans, molluscs  Fish products, fish in crumbs  Amphibians and reptiles |
| Eggs | ‘09’ Eggs and egg products |  |
| Plant-based foods | | |
| Potatoes | ‘01’ Potatoes and other tubers |  |
| Vegetables | ‘02’ Vegetables | Unclassified, mixed salad/vegetables  Leafy vegetables (except cabbages)  Fruiting vegetables  Root vegetables  Cabbages  Mushrooms  Grain and pod vegetables  Leek, onion, garlic  Stalk vegetables, sprouts |
| Legumes | ‘03’ Legumes |  |
| Fruits | ’04-01’ Fruits  ’04-03’ Olives | Unclassified, mixed fruits, fruit compote  Fruits  Fruit compote |
| Nuts and seeds | ’04-02’ Nuts, peanuts, seeds and nut spread | Unclassified nuts and seeds (+ nut spread  Nuts, peanuts, seeds  Peanut butter, nut/seeds spread |
| Cereals (Refined grains & Whole grains^a^) | ‘06’ Cereals and cereal products | Unclassified and combined cereal products  Flours, starches, flakes, and semolina  Pasta, rice, other grain  Bread, Crispbread, rusks  Breakfast cereals  Dough and pastry (plain puff, short-crust, etc.) |
| Beverages | | |
| Fruit and vegetable juice | ‘13-01’ Fruit and vegetable juice |  |
| Soft drinks | ‘13-02’ Carbonated/soft/isotonic drinks, diluted |  |
| Coffee and tea | ‘13-03’ Coffee, tea and herbal tea | Unclassified and combined coffee/tea drin  Coffee  Tea  Herbal tea  Chicory, substitutes |
| Water | ‘13-04’ Water |  |
| Alcoholic beverages | ‘14’ Alcoholic beverages | Unclassified, cocktails, punches  Wine, cider, fruit wines  Fortified wines (sherry,porto,vermouth,..  Beer  Spirits, brandy  Aniseed drinks (pastis,..)  Liqueurs |
| Miscellaneous | | |
| Sweets and snacks | ‘11’ Sugar and confectionery  ‘12’ Cakes and sweet biscuits  ‘18’ Savoury snacks. | Unclassified or combined confectionery items  Unclassified and other sugar, honey, jam,  Sugar  Jam, jelly, marmelade  Honey  Other sweet spread  Sweet sauce, sweet topping for desserts  Syrup (incl. from can and for beverages)  Unclassified and other chocolate confecti  Chocolate tablet  Chocolate candy bars  Chocolate spread and chocolate powder  Chocolate confectionery  Confectionery non chocolate  Unclassified and combined cakes, biscuits  Cakes, pies, pastries, puddings (non-milk  Dry cakes, sweet biscuits |
| Fats and oils | ‘10’ Fats and oils | Unclassified and combined fats  Vegetable oils  Butter  Margarines and cooking fats  Other animal fats (including fish oils) |
| Broth, sauces and condiments | ‘15’ Condiments, spices, sauces and yeast and ‘16’ Soups and stocks | Unclassified or combined condiments and spices  Other and mixed sauces  Tomato sauces  Dressing sauces, mayonnaises and similar  Mayonnaise based spreads  Yeast  Spices, herbs and flavourings  Unclassified and combined condiments  Vinegar |
| Other | ‘17’ Miscellaneous (excluding ’17-01’ Vegetarian products/dishes); | Unclassified or combined miscellaneous food  Unclassified and combined dietetic products  Artificial sweeteners  Meal substitutes  Insects |

^a^ Grouping into refined or whole grains was done based on the cut-off value of the Dutch dietary guidelines: cereals and cereal products that exist of at least 25% whole grains and cereal bran (Dutch Health Counsil. 2015). The script is available on request from the authors.

## References

Dutch Health Counsil. (2015). Cereals and cereal products - Background Document for Dutch Dietary Guidelines 2015; Granen en graanproducten - Achtergronddocument bij Richtlijnen goede voeding 2015. The Hague, The Netherlands, Dutch Health Counsil.

# Supplement 3

Formulas used to calculate predicted values for meat quartile 1. Similar calculations are performed for other meat quartiles.

/** Calculate predicted value log_amount**/

Log_Amount_Q1_M=a2+b2***10.2**+X[,**2**];

Log_Amount_Q1_F=a2+b2***10.2**+c2+d2***10.2**+X[,**2**]; *0=male, 1=female;

Explanation variables:

- Log_amount_Q1_M: the predicted log amount of the respective food group (that is used as dependent variable) of meat quartile 1 for males.
- Log_Amount_Q1_F: the predicted log amount of the respective food group (that is used as dependent variable) of meat quartile 1 for females.
- A2: intercept for consumption of food group for the amount part of the model
- B2: regression coefficient for meat for amount part of the model
- 10.2: mean amount of meat consumption in meat quartile 1
- C2: coefficient for sex for the amount part of the model
- D2: coefficient for the interaction term between meat and sex for the amount part of the model
- X: matrix of 2 by 10000 with simulated random effects for probability (column 1) and amount (column 2) part of the model
- Note: these calculations are repeated for the other meat quartiles.

/** Backtransforming log_amount to amount**/

Amount_Q1_M=exp(log_Amount_Q1_M+(**0.5***SD^2^));

Amount_Q1_F=exp(log_Amount_Q1_F+(**0.5*** SD^2^));

Explanation variables:

- Amount_Q1_M: the predicted amount of the respective food group (that is used as dependent variable) of meat quartile 1 for males.
- SD: standard deviation of the day-to-day variation

/** Calculate predicted value log_probability**/

Log_Probability_Q1_M=a1+b1***10.2**+X[,**1**];

Log_Probability_Q1_F=a1+b1***10.2**+c1+d1***10.2**+X[,**1**];

Explanation variables:

- Log_Probability_Q1_M: the predicted log probability of the respective food group (that is used as dependent variable) of meat quartile 1 for males.
- Log_Probability_Q1_F: the predicted log probability of the respective food group (that is used as dependent variable) of meat quartile 1 for females.
- A1: intercept for consumption of food group for the probability part of the model
- B1: regression coefficient for meat for probability part of the model
- 10.2: average amount of meat consumption in meat quartile 1
- C1: coefficient for sex for probability part of the model
- D1: coefficient for interaction term between meat and sex for probability part of the model
- X: matrix of 2 by 10000 with simulated random effects for probability (column 1) and amount (column 2) part of the model
- Note: these calculations are repeated for the other meat quartiles.

/** Backtransforming log_probability to probability**/

Probability_Q1_M_X=exp(log_Probability_Q1_M);

Probability_Q1_M=Probability_Q1_M_X/(**1**+Probability_Q1_M_X);

Explanation variables:

- Probability_Q1_M_X: odds of the respective food group (that is used as dependent variable) of meat quartile 1 for males.
- Probability_Q1_M: the predicted probability of the respective food group (that is used as dependent variable) of meat quartile 1 for males.

/** Multiplying amount by probability to obtain final predicted value**/

P_A_Q1_M=Amount_q1_m#Probability_q1_m;

P_A_Q1_F=Amount_Q1_F#Probability_Q1_F;

/** calculate the sample mean **/

SampleMean_P_A_Q1_M = P_A_Q1_M[:, ];

SampleMean_P_A_Q1_F = P_A_Q1_F[:, ];

SampleMean_Amount_Q1_M = Amount_Q1_M[:, ];

SampleMean_Amount_Q1_F = Amount_Q1_F[:, ];

SampleMean_Probability_Q1_M = Probability_Q1_M[:, ];

SampleMean_Probability_Q1_F = Probability_Q1_F[:, ];

# Supplement 5

Supplemental figure 5a. Consumption of vegetables presented by mean total meat consumption per quartile, gender, and age. The predicted quantity (Probability*Amount) (gram/2,000 kcal) is the modelled amount among users (Amount), times the modelled probability of consumption (Probability) derived from the Dutch National Food Consumption Survey 2012-2016.

Supplemental figure 5b. Consumption of nuts and seeds presented by mean total meat consumption per quartile, gender, and age. The predicted quantity (Probability*Amount) (gram/2,000 kcal) is the modelled amount among users (Amount), times the modelled probability of consumption (Probability) derived from the Dutch National Food Consumption Survey 2012-2016.

Supplemental figure 5c. Consumption of refined grains presented by mean total meat consumption per quartile, gender, and age. The predicted quantity (Probability*Amount) (gram/2,000 kcal) is the modelled amount among users (Amount), times the modelled probability of consumption (Probability) derived from the Dutch National Food Consumption Survey 2012-2016.

Supplemental figure 5d. Consumption of sweets and snacks presented by mean total meat consumption per quartile, gender, and age. The predicted quantity (Probability*Amount) (gram/2,000 kcal) is the modelled amount among users (Amount), times the modelled probability of consumption (Probability) derived from the Dutch National Food Consumption Survey 2012-2016.

Supplemental figure 5e. Consumption of **whole grains** presented by mean total meat consumption per quartile, gender, and age. The predicted quantity (Probability*Amount) (gram/2,000 kcal) is the modelled amount among users (Amount), times the modelled probability of consumption (Probability) derived from the Dutch National Food Consumption Survey 2012-2016.
