# Supplementary material for: Low Meat Consumption in the Netherlands Is Associated With Higher Intake of Fish, Nuts, Seeds, Cheese, Sweets, and Snacks: Results From a Two-Part Model
Source: Front Nutr. 2022 Jan 26;8:741286. doi: 10.3389/fnut.2021.741286 (PMC8825789; doi:10.3389/fnut.2021.741286)
Supplement: Supplementary file 3 [file Presentation_1.pptx]

## Slide 1
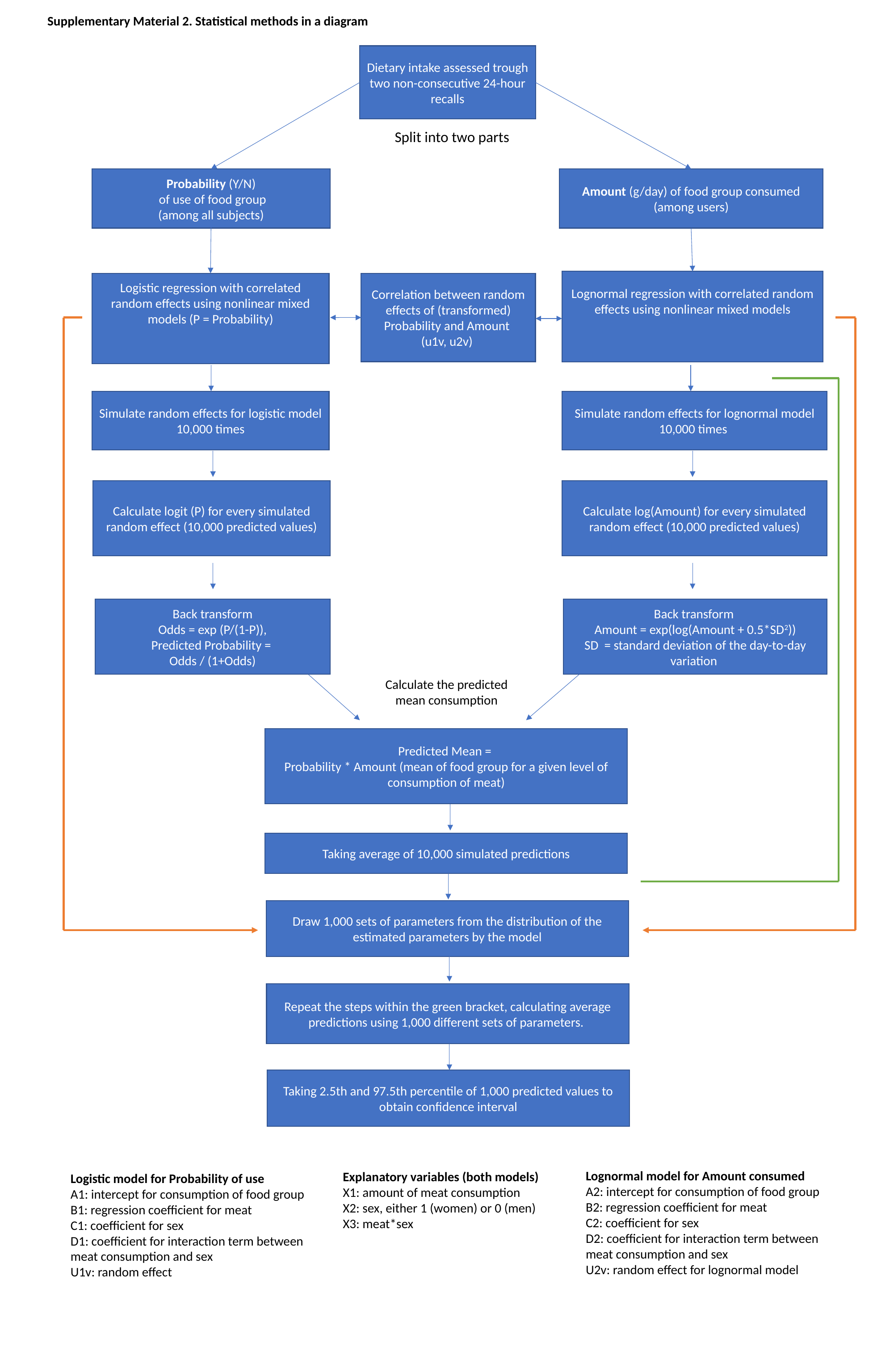

Supplementary Material 2. Statistical methods in a diagram
Dietary intake assessed trough two non-consecutive 24-hour recalls
Split into two parts
Probability (Y/N)
 of use of food group
(among all subjects)
Amount (g/day) of food group consumed
(among users)
Lognormal regression with correlated random effects using nonlinear mixed models
Logistic regression with correlated random effects using nonlinear mixed models (P = Probability)
Correlation between random effects of (transformed) Probability and Amount
(u1v, u2v)
Simulate random effects for logistic model 10,000 times
Simulate random effects for lognormal model 10,000 times
Calculate logit (P) for every simulated random effect (10,000 predicted values)
Calculate log(Amount) for every simulated random effect (10,000 predicted values)
Back transform
Odds = exp (P/(1-P)),
Predicted Probability =
Odds / (1+Odds)
Back transform
Amount = exp(log(Amount + 0.5*SD2))
SD = standard deviation of the day-to-day variation
Calculate the predicted mean consumption
Predicted Mean =
Probability * Amount (mean of food group for a given level of consumption of meat)
Taking average of 10,000 simulated predictions
Draw 1,000 sets of parameters from the distribution of the estimated parameters by the model
Repeat the steps within the green bracket, calculating average predictions using 1,000 different sets of parameters.
Taking 2.5th and 97.5th percentile of 1,000 predicted values to obtain confidence interval
Lognormal model for Amount consumed
A2: intercept for consumption of food group
B2: regression coefficient for meat
C2: coefficient for sex
D2: coefficient for interaction term between meat consumption and sex
U2v: random effect for lognormal model
Explanatory variables (both models)
X1: amount of meat consumption
X2: sex, either 1 (women) or 0 (men)
X3: meat*sex
Logistic model for Probability of use
A1: intercept for consumption of food group
B1: regression coefficient for meat
C1: coefficient for sex
D1: coefficient for interaction term between meat consumption and sex
U1v: random effect
